# COVID‐19 coagulopathy – what should we treat?

**DOI:** 10.1113/EP089404

**Published:** 2022-06-22

**Authors:** Pratima Chowdary

**Affiliations:** ^1^ Katharine Dormandy Haemophilia and Thrombosis Centre Royal Free Hospital London UK; ^2^ Cancer Institute University College London London UK

**Keywords:** anticoagulants, ARDS, coagulation, coronavirus, COVID‐19, COVID coagulopathy, fibrinolytics, hypercoagulability, pulmonary microvascular thrombosis, thromboinflammation

## Abstract

**New Findings:**

- **What is the topic of this review?**
  Overview of the coagulation abnormalities, including elevated D‐dimers widely reported with COVID‐19, often labelled as COVID coagulopathy.
- **What advances does it highlight?**
  The review highlights the changes in bronchoalveolar haemostasis due to apoptosis of alveolar cells, which contributes to acute lung injury and acute respiratory distress syndrome; the pathophysiological mechanisms, including endothelial dysfunction and damage responsible for thrombosis of pulmonary microcirculation and potential contribution to the hypoxaemia of COVID‐19 acute lung injury; and changes in coagulation proteins responsible for the hypercoagulability and increased risk of thrombosis in other venous and arterial beds. The rationale for anticoagulation and fibrinolytic therapies is detailed, and potential confounders that might have led to less than expected improvement in the various randomised controlled trials are considered.

**Abstract:**

Coronavirus disease 19 (COVID‐19) causes acute lung injury with diffuse alveolar damage, alveolar–capillary barrier disruption, thrombin generation and alveolar fibrin deposition. Clinically, hypoxaemia is associated with preserved lung compliance early in the disease, suggesting the lack of excessive fluid accumulation typical of other lung injuries. Notably, autopsy studies demonstrate infection of the endothelium with extensive capillary thrombosis distinct from the embolic thrombi in pulmonary arteries. The inflammatory thrombosis in pulmonary vasculature secondary to endothelial infection and dysfunction appears to contribute to hypoxaemia. This is associated with elevated D‐dimers and acquired hypercoagulability with an increased risk of deep vein thrombosis. Hypercoagulability is secondary to elevated plasma tissue factor levels, von Willebrand factor, fibrinogen, reduced ADAMTS‐13 with platelet activation and inhibition of fibrinolysis. Multi‐platform randomised controlled studies of systemic therapeutic anticoagulation with unfractionated and low molecular mass heparins demonstrated a survival benefit over standard care with full‐dose anticoagulation in patients with non‐severe disease who require supplemental oxygen, but not in severe disease requiring ventilatory support. Late intervention and the heterogeneous nature of enrolled patients can potentially explain the apparent lack of benefit in severe disease. Improvement in oxygenation has been demonstrated with intravenous fibrinolytics in small studies. Inhaled anticoagulants, thrombolytic agents and non‐specific proteolytic drugs in clinical trials for decreasing alveolar fibrin deposition might benefit early disease. Essentially, COVID‐19 is a multi‐system disorder with pulmonary vascular inflammatory thrombosis that requires an interdisciplinary approach to combination therapies addressing both inflammation and intravascular thrombosis or alveolar fibrin deposits to improve outcomes.

## 1 INTRODUCTION

Severe acute respiratory syndrome coronavirus 2 (SARS‐CoV‐2), responsible for the pandemic of novel coronavirus disease 2019 (COVID‐19), is a self‐limiting illness in the majority. Around a fifth of unvaccinated patients developed a severe disease, of whom a quarter progressed to critical illnesses (Wu & McGoogan, 2020), including acute respiratory distress syndrome (ARDS), multi‐organ failure and death (Yang et al., 2020).

SARS‐CoV‐2 is a single‐stranded RNA virus enveloped in a lipid bilayer with embedded structural proteins, including the spike protein. The virus primarily infects alveolar type II cells, which secrete pulmonary surfactant and are the progenitor cells for alveolar type I cells (Lamers & Haagmans, 2022). Alveolar type I cells, which mediate gas exchange, cover 95% of the internal surface of the alveolus and share a basement membrane with the pulmonary vascular endothelial cells. Cellular entry is initiated by the spike protein interacting with the angiotensin‐converting enzyme 2 (ACE2) receptor via the receptor‐binding domain. Entry is facilitated by other proteases on the cell surface that cleave the spike protein. ACE2 in normal lungs protects against lung injury (Samavati & Uhal, 2020).

In severe COVID‐19 disease, the diffuse alveolar damage disrupts the alveolar–capillary barrier, with the accumulation of plasma rich in albumin and fibrinogen, fibrin deposits, and cellular debris, and interstitial infiltration with inflammatory cells. Notably, autopsy studies have demonstrated the presence of the virus in endothelial cells with endothelial cell apoptosis and thrombi in the capillaries (Ackermann et al., 2020; Varga et al., 2020). The mechanisms of entry into vascular endothelial cells are under investigation by several groups. It is known that quiescent vascular endothelial cells do not demonstrate ACE2 receptors, but infection of epithelial cells has been associated with upregulation of ACE2 expression on vascular endothelial cells suggesting a potential mechanism of cell entry, which needs confirmation in vivo (Liu et al., 2021).

The innate immune response is the first line of defence for any viral infection. It is typically rapid and coordinated, but a dysregulated and excessive immune response, that is, the cytokine storm or cytokine release syndrome, can contribute to additional tissue damage (Teijaro, 2017). Cytokine release syndrome is characterised by increased levels of pro‐inflammatory cytokines and chemokines (Channappanavar & Perlman, 2017; Costela‐Ruiz et al., 2020). The relative contribution of the viral cytotoxicity and cytokine release syndrome to the diffuse alveolar damage is not well elucidated. Indeed transcriptional profiling of lower respiratory tract specimens demonstrates a reduced pro‐inflammatory gene expression in COVID‐19 ARDS compared to ARDS due to other causes (Sarma et al., 2021). Nevertheless, the reduction in mortality seen following immunomodulation with steroids and Janus kinase‐2 inhibitors confirms the significant contribution of inflammation to mortality (Horby et al., 2021; Kalil et al., 2021).

This review aims to provide an overview of the haemostatic changes both in the alveolar and vascular space, the impact on outcomes and the potential targets for therapeutic intervention with antithrombotic agents.

## 2 COAGULOPATHY – EXTRAVASCULAR OR INTRAVACULAR

### 2.1 Definition

The term ‘coagulopathy’ in common use has no agreed definition. Broadly, it is used to describe coagulation derangements secondary to multi‐system disorders that present as abnormalities of routine coagulation tests. Clinically, the patients may be asymptomatic or present with bleeding or clotting (thrombosis) issues. The abnormalities of routine coagulation tests commonly seen include a prolonged prothrombin time, shortened or prolonged activated partial thromboplastin time (aPTT) or abnormal thrombin time. These changes can be associated with the abnormalities of D‐dimers, a marker of cross‐linked fibrin and clot turnover, and fibrinogen, an acute‐phase protein.

### 2.2 Extravascular coagulopathy

Acute lung injury or ARDS is characterised by extravascular fibrin deposition localised to the alveolar compartment following alveolar cell damage and disruption of the alveolar–capillary barrier. This fibrin deposition is essential for restoring the alveolar–capillary barrier and subsequent repair (Idell, 2003). It is pertinent to recall that coagulation is a host defence mechanism responsible for creating a temporary barrier to maintain cutaneous and/or mucosal surface integrity in the event of a physical disruption. In addition to the immediate protection, the fibrin deposition in the extracellular matrix acts as a scaffold for tissue repair by supporting epithelial cell and fibroblast proliferation, migration and growth.

Fibrin deposition is facilitated by in situ thrombin generation due to local overexpression of tissue factor on the injured cell surfaces and procoagulants in the leaked plasma. Elevated circulating extracellular vesicle tissue factor levels observed in COVID‐19 correlate with mortality and probably originate from damaged pulmonary tissue. Of note, in vitro studies of SARS‐CoV demonstrated an upregulation of mRNA for all three subunits of fibrinogen secondary to an expression of one of the SARS‐CoV proteins (Tan et al., 2005). Further, in ARDS, alveolar fibrinolysis is inhibited due to decreased expression of urokinase plasminogen activator and increased expression of plasminogen activation inhibitor‐1 (Hofstra et al., 2008; Idell et al., 1992).

The binding of surfactant to cross‐linked fibrin decreases its activity, contributing to a loss of function and alveolar collapse (Seeger et al., 1993). In addition, the incorporation of surfactant into the developing clot increases resistance to lysis. In this context, in vitro experiments with fibrinolytics, particularly plasminogen activators, have demonstrated the restoration of surfactant activity (Günther et al., 2001; Schuliga et al., 2018). In COVID‐19, the primary infection of type II alveolar cells is likely to significantly impair surfactant generation.

Fibrin formation within the lungs can also initiate a vicious cycle of tissue damage. Fibrin deposition, whilst beneficial in the restitution of the alveolar–capillary barrier following acute lung injury, can set the scene for pathological fibrosis if persistent (Schuliga et al., 2018). Neutrophils localised to the clot through integrin binding sites within the fibrinogen molecule (Cooper et al., 1988) can exacerbate inflammation (Grommes & Soehnlein, 2011). Further dysregulation of neutrophil extracellular trap formation in COVID‐19 has been demonstrated to contribute to tissue damage (Thierry & Roch, 2020). Post‐mortem studies of COVID‐19 ARDS lungs demonstrated a marked lymphocytic infiltrate when patients died early in their disease, but deaths in advanced stages were characterised by extensive intra‐alveolar fibrin accumulation (fibrin ‘balls’), suggesting inadequate clearance (Copin et al., 2020).

Evolving evidence suggests that COVID‐19 ARDS is distinct from conventional ARDS. Clinically, the ARDS of COVID‐19 shows a dissociation between hypoxaemia and respiratory mechanics, with relatively well‐preserved compliance, despite severe hypoxaemia. This initial hypoxaemia may be related to the early loss of surfactant due to infection of type II alveolar cells and local fibrin deposition. As the disease progresses, the clinical picture is in keeping with conventional ARDS with decreasing compliance due to increased fluid in the lung (Gattinoni et al., 2020). The alveolar fluid accumulation typical of conventional ARDS is less intense and appears later, potentially representing a severe inflammatory response triggered by a potential combination of viral infection, fibrin deposition and tissue damage by neutrophils and macrophages.

### 2.3 Intravascular coagulopathy

Blood coagulation abnormalities in the form of significantly elevated D‐dimer were noted early in the clinical characterisation of COVID‐19 and have been associated with higher mortality (Tang et al., 2020; Thachil et al., 2020). The first suggestion that D‐dimers might represent more than endothelial dysfunction was the demonstration of viral inclusions in the endothelial cells in addition to perivascular inflammation suggesting primary infection of the endothelial cells, labelled as endotheliitis (Varga et al., 2020). Post‐mortem investigation of vasculature in patients with influenza (A/H1N1) and COVID‐19 demonstrated a differential distribution and density of thrombi across the pulmonary vasculature (Ackermann et al., 2020). Fibrin thrombi in alveolar capillaries were nine times more common in COVID‐19 compared to influenza patients (mean ± SD number of distinct thrombi/cm^2^ of vascular lumen area, 159 ± 73 vs. 16 ± 16), with the latter demonstrating a more significant number of thrombi in post‐capillary venules of <1 mm (12 ± 14 vs. 35 ± 16). In pulmonary arteries of 1–2 mm, thrombi were variably present in around half of the patients in both groups with no difference in the density.

Several groups have reported clinically significant thrombosis in pulmonary arteries on imaging in COVID‐19 patients (Patel et al., 2020). It has been proposed that the pulmonary thrombi in COVID‐19 represent *de novo* thrombus formation, secondary to local inflammation instead of having an embolic origin (Ackermann et al., 2020; Cattaneo et al., 2020), and the term ‘pulmonary intravascular coagulopathy’ has been coined to describe this (McGonagle et al., 2020).

In a large case series of 283 deceased COVID‐19 patients, pneumonia and/or diffuse alveolar damage was the most common cause of death (73.6%), with thrombosis detected in 39.2% and pulmonary embolism (PE) in 22.1% (Fitzek et al., 2021). Indeed, in a small randomised study terminated prematurely, the occurrence of PE was not associated with concomitant deep vein thrombosis (DVT), confirming the *de novo* nature of the pulmonary thrombi (Morici et al., 2021). A cohort study showed an increasing prevalence of PE with increasing D‐dimers, with a 10‐fold elevation strongly predictive of PE (Perera et al., 2021).

In addition to local changes in the pulmonary vasculature, there is accumulating evidence of systemic hypercoagulability that increases the risk of deep vein thrombosis with secondary embolisation. Consistent findings contributing to the hypercoagulability include elevated von Willebrand factor secondary to endothelial dysfunction (Goshua et al., 2020); elevated fibrinogen and FVIII, both acute‐phase proteins (Gabay & Kushner, 1999); reduced thrombomodulin (Goshua et al., 2020); and reduced ADAMTS‐13 with altered von Willebrand factor/ADAMTS‐13 ratio (Favaloro et al., 2021). All abnormalities typically are more prominent in patients in intensive care requiring ventilatory support than in patients on the ward with less severe disease.

Other markers of endothelial dysfunction observed include elevated levels of syndecan and tissue factor protein inhibitor, both associated with the endothelial glycocalyx and mediating local anticoagulant activity in the microcirculation. In a small study comparing COVID‐19 positive and negative patients in intensive care, the COVID‐19 group had a higher and more persistent elevation of syndecan‐1 (Fraser et al., 2020). Loss of endothelial glycocalyx has been reported due to the induction of matrix metalloproteases by cytokines (Masola et al., 2021).

Cohort studies have not demonstrated a marked increase in thrombin generation despite the elevated FVIII. This may be related to the lack of reduction in the natural anticoagulants antithrombin (AT), protein C and protein S (von Meijenfeldt et al., 2021), with reductions only seen in patients with endstage disease with secondary complications.

It is now firmly established that COVID‐19 is associated with an excess of arterial and venous thrombotic events (VTE) due to a combination of pulmonary vascular inflammatory thrombosis and systemic hypercoagulability. However, the exact prevalence and contribution to mortality are uncertain. One meta‐analysis reports a pooled frequency of PE in COVID‐19 in the emergency department, general wards and intensive care at 17.9%, 23.9% and 48.6%, respectively, and D‐dimer cut‐off levels used to identify patients vary from 2‐ to 10‐fold higher than the upper limit of normal (Kwee et al., 2021). Importantly, another meta‐analysis of the VTE risk factors demonstrated no association with traditional risk factors for VTE (e.g. obesity, previous VTE) but showed an association between VTE and D‐dimer peak, length of hospitalisation and need for intubation (Lobbes et al., 2021).

### 2.4 COVID coagulopathy and D‐dimers

The term ‘COVID coagulopathy’ has been coined to draw attention to the various coagulation abnormalities seen in this disorder, particularly high D‐dimers. At its core, it is an inflammatory coagulopathy with a normal to borderline prolonged prothrombin time, normal or shortened aPTT, elevated D‐dimers, increased fibrinogen and normal platelet count along with microvascular thrombi (Jackson et al., 2019). Elevated D‐dimers in COVID‐19 have multiple sources, including endothelial dysfunction, clot turnover from *de novo* pulmonary microvascular thrombi and DVT with and without embolism. Indeed, elevated D‐dimers appear to predict pulmonary vascular thrombi (Lobbes et al., 2021; Perera et al., 2021), and imaging can identify subclinical presentations (Patel et al., 2020). However, the specificity of conventional thresholds of D‐dimers for predicting VTE in this context is low (Chen et al., 2020; Zhan et al., 2021), and higher thresholds are appropriate.

Some COVID‐19 coagulopathy features are also seen with consumptive coagulopathy, that is, disseminated intravascular coagulation with microvascular thrombi and consumption of the various coagulation factors (Taylor et al., 2001). Disseminated intravascular coagulation presents with a variably prolonged prothrombin time, prolonged aPTT, elevated D‐dimers, normal or reduced platelet count, and normal or reduced fibrinogen (Gando et al., 2013; Toh & Hoots, 2007). Indeed radiation exposure in pigs causes both a consumptive coagulopathy and pulmonary fibrosis, unlike COVID‐19, which is complicated by inflammatory coagulopathy and pulmonary fibrosis (Krigsfeld et al., 2014).

## 3 COVID COAGULOPATHY – CLINICAL IMPLICATIONS AND THERAPEUTIC STRATEGIES

**FIGURE 1:**
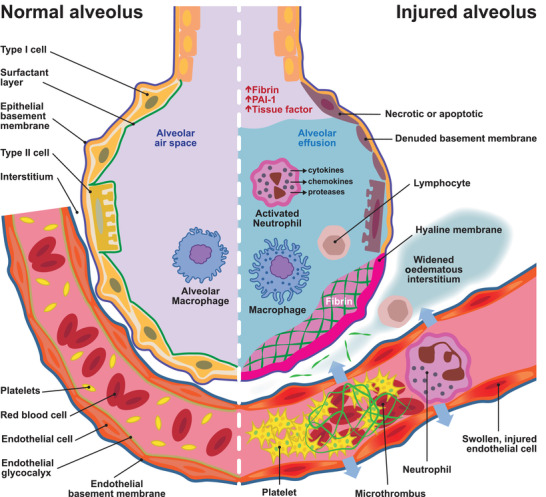
COVID‐19 ARDS – coagulation changes in the alveoli and pulmonary microcirculation. PAI‐1: plasminogen activation inhibitor‐1

Based on the pathophysiology of COVID coagulopathy, potential targets for intervention are either alveolar thrombi or vascular thrombi (Figure 1). Therapeutic interventions can decrease the risk of clot formation through anticoagulants and antiplatelet agents or reduce clot burden through direct clot lysis with a fibrinolytic agent. Clot burden can also be reduced indirectly with anticoagulation by reducing clot propagation and facilitating endogenous clot lysis.

### 3.1 Extravascular coagulopathy – fibrinolytics and proteolytics for alveolar thrombi

As described, alterations in bronchoalveolar haemostasis are a host defence mechanism that facilitates control of infection, tissue repair and recovery. However, if the clearance mechanisms are overwhelmed or impaired, a fibrotic reaction can cause slow or no recovery. Alveolar thrombi undergo spontaneous lysis with the regeneration of alveolar type I cells. Impaired lysis is conceivable in the context of slow regeneration of the type I cells or if the clot burden is high or resistant to lysis. The aim of treating alveolar thrombi is to improve oxygenation, and therapeutic interventions via the inhaled route to decrease clot burden include fibrinolytic drugs like streptokinase or recombinant tissue plasminogen activator (rtPA), anticoagulants like unfractionated heparin (UFH) and non‐specific proteolytic drugs.

Accumulated data from clinical and animal models suggest that both intravenous and inhaled thrombolytic drugs in acute lung injury are associated with increased oxygenation and improved mortality rates due to improved fibrin clearance from the alveolar space (Hofstra et al., 2013). A meta‐analysis of 22 studies of fibrinolytics in animal models of acute lung injury demonstrated significantly increased fibrinolytic activity in plasma and bronchoalveolar lavage fluid when administered by inhalation or intravenously. Further, there was an apparent reduction in inflammation (alveolar neutrophils) when rtPA was directly administered to the lung as a nebulised therapy (Foley, 2017; Liu et al., 2018).

Following an initial case report demonstrating the success of nebulised rtPA in ARDS (Gram et al., 1999), a randomised controlled trial of nebulised streptokinase showed improved survival compared to standard of care in patients with ARDS, with the restoration of oxygenation to normal levels within 3 days (Abdelaal Ahmed Mahmoud et al., 2020). Inhalation of rtPA used in patients with plastic bronchitis, a disorder characterised by fibrin bronchial casts, was not associated with excess bleeding (Lackowski et al., 2010).

Drugs that are undergoing clinical trials include inhaled anticoagulants and non‐specific proteolytic drugs. The anticoagulant of choice for inhalation has been UFH. The clinical trials currently recruiting vary in their dosing regimen and duration of treatment and often include patients on mechanical ventilation. UFH is an indirect anticoagulant dependent on AT for total activity. The AT in alveoli appears to originate from the plasma exudate. UFH can affect the clot structure in the absence of AT (Yeromonahos et al., 2012). Other drugs that aim to facilitate the clearance of mucus and debris include N‐acetylcysteine (Shi & Puyo, 2020) and dornase alfa, which facilitate mucus clearance by cleaving the neutrophil‐derived extracellular double‐stranded DNA (Gavriilidis et al., 2022) and mucolytic combinations in clinical trials (N‐acetylcysteine and bromelin).

### 3.2 Intravascular coagulopathy – antiplatelets, anticoagulants and fibrinolytics

In COVID‐19, endothelial dysfunction due to proximity to the alveolar inflammation triggers pulmonary capillary vessel thrombosis, which may help localise the infection. Primary infection of endothelial cells accentuates this, and increasing severity results in the progression of the thrombosis from the microcirculation to the small vessels and subsegmental pulmonary arteries. Management of vascular thrombi is well‐established, and interventions like fibrinolytics either clear pulmonary vascular thrombi directly or indirectly with anticoagulants and antiplatelet agents. The archetype of inflammation‐induced thrombosis is Behçet's syndrome, where the role of anticoagulation in addition to immunomodulation is still the subject of debate (Ahn et al., 2008; Emmi et al., 2018). Pulmonary vascular thrombosis of COVID‐19 is typical of inflammatory thrombosis and may be resistant to standard treatment. Further, inflammation‐induced acquired hypercoagulability increases the risk of deep vein thrombosis with embolisation to pulmonary vasculature (main vessel, lobar, segmental and subsegmental pulmonary arteries). Anticoagulants and antiplatelets can decrease the risk of thrombosis across the vasculature by decreasing systemic hypercoagulability.

### 3.3 Anticoagulants and antiplatelets

Following the initial publication of potential survival benefits with prophylactic low molecular mass heparin (Tang et al., 2020), numerous randomised controlled clinical trials of the anticoagulants and antiplatelet agents have been conducted to ascertain survival benefits and development of thrombotic events. A review of clinical trials.gov at the time of this publication showed around 100 trials with anticoagulants, including unfractionated and low molecular mass heparins, direct oral anticoagulants (rivaroxaban, apixaban, edoxaban, dabigatran), another 50 with antiplatelet agents (aspirin, clopidogrel, dipyridamole) and around 10 with antifibrinolytic agents (rtPA). In addition, clinical trials have also attempted a risk–benefit analysis of different intensities of anticoagulation. Of note, most trials appear to restrict evaluation to immediate outcomes, with no data collected on long‐term outcomes.

Multi‐platform randomised controlled studies of systemic therapeutic anticoagulation with unfractionated and low molecular mass heparins in COVID‐19 demonstrated a survival benefit with full‐dose anticoagulation in patients with non‐severe disease requiring supplemental oxygen compared to standard of care (Lawler et al., 2021) but not in severe disease requiring ventilatory support, invasive and non‐invasive ventilation (Goligher et al., 2021). Similarly, the use of antiplatelet aspirin was not associated with any survival benefit (RECOVERY, 2021).

However, there are several challenges in interpreting this evidence, and some have been reviewed (Connors et al., 2021). One issue with a significant impact on the antithrombotic clinical trial outcomes is related to the inclusion criteria for the trials. The pragmatic nature of trials meant that the presence of COVID‐19 determining eligibility and disease severity dictated treatment with no data on underlying clot burden. This issue is further compounded by the heterogeneity around the measured outcomes in clinical trials (Table 1). Another confounder is the timing of the initiation of anticoagulation in relation to the natural history of the disease, potentially explaining the paradoxical results seen in multiplatform studies. Improved survival observed in non‐severe disease but not in severe disease suggests that anticoagulation earlier in the disease progression is able to control the thrombosis. As COVID‐19 pulmonary vascular thrombosis is an inflammatory thrombosis, it is conceivable that in severe disease, anticoagulation alone is unlikely to facilitate endogenous lysis without anti‐viral and immunomodulating therapies to decrease tissue damage. The risk–benefit analysis for therapeutic anticoagulation is further distorted by using the International Society of Haemostasis and Thrombosis (ISTH) criteria for bleed event categorisations (Schulman & Kearon, 2005). The ISTH criteria were developed for an ambulant population with a low risk of bleeding and mortality, unlike severe COVID‐19, with a high risk of mortality, and the appropriateness of ISTH major bleeding definition for this group is questionable.

**TABLE 1:** Common outcomes used in clinical trials of anticoagulants, anti‐fibrinolytics and antiplatelet agents

Decreased need for oxygen support and ventilation Decreased duration of oxygen support Decreased need for ventilation Decrease in symptomatic venous and arterial thrombosis Fewer symptomatic events Fewer asymptomatic events Overall decrease in mortality Mortality at day 30 and day 90 Reduction in disease severity Change in WHO ordinal scale or similar scale Reduction in length of hospitalisation Composite endpoints	

Nevertheless, the concern is the assumption that adjuvant therapy, in this instance anticoagulation, might increase survival. The development of thrombosis in the pulmonary vasculature and deep veins is a complication of the underlying disease process. Interventions for the complication without addressing the underlying disease are unlikely to yield the desired results in patients with severe disease. Therefore, the lack of apparent efficacy cannot be ascribed solely to lack of effect. Indeed, it may indicate that combination therapies targeting inflammation and thrombosis might be needed.

A reduction in hypercoagulability through prophylactic anticoagulation can decrease the risk of deep vein thrombosis and embolisation. There is also an active discussion about the use of D‐dimers for stratification of patients for prophylactic/therapeutic anticoagulation. Certain investigators have suggested anticoagulants in every patient with elevated D‐dimers, and others have recommended a restriction to patients with multiple comorbidities and at high risk of developing severe disease or patients hospitalised with the infection. It is important to recall that D‐dimers represent thrombus turnover and, therefore, coagulation activation but are not hypercoagulability markers. The underlying question that is yet to be answered unequivocally is the magnitude of the impact of antithrombotic treatment on survival and incidence of symptomatic thrombotic events.

### 3.4 Fibrinolytics

Since the first demonstration of improvement in oxygenation following intravenous rtPA in ARDS associated with trauma (Hardaway, 2006), there has been an interest in investigating this treatment modality, but concerns about bleeding have tempered clinical studies. The pathophysiological rationale for fibrinolytic therapy has been extensively reviewed (Barrett et al., 2020; Whyte et al., 2020). Several case series in COVID‐19 have shown improvement in oxygenation with intravenous rtPA in COVID‐19 patients with severe ARDS (Arachchillage et al., 2020; Savioli et al., 2020; Wang et al., 2020), but the impact on morbidity and mortality is unclear. A pilot phase 3 study demonstrated the safety of intravenous rtPA in COVID‐19 ARDS, with an improvement in oxygenation at 6 h through 7 days, confirming the contribution of pulmonary microthrombi to the pathogenesis of the hypoxaemia of COVID‐19 ARDS (Barrett et al., 2021). Although oxygenation improved, the study was not powered to demonstrate clinical improvements. Importantly, the study showed the safety of the rtPA in this cohort to facilitate larger phase 3 randomised studies, which are currently ongoing.

## 4 CONCLUSION

Coagulation is one of the host defence mechanisms to control infection and inflammation. There is now convincing evidence that hypoxaemia following SARS‐CoV‐2 infection is due to alveolar and endothelial damage with extensive capillary thrombi. Further, patients can also develop deep vein thrombosis with embolisation secondary to hypercoagulability and immobility. Elevated D‐dimers can serve as prognostic markers and identify patients who might benefit from imaging studies to exclude pulmonary vascular thrombosis. The detection of pulmonary vascular thrombi might require a higher D‐dimer threshold for imaging than conventional VTE.

Whilst anti‐viral interventions and immunomodulatory agents have demonstrated clinical benefit, clinical trials of anticoagulation and fibrinolytic agents have been more challenging to conduct and interpret. Some of the challenges are related to the fact that infection has determined trial entry rather than the presence of thrombosis. However, a role for antithrombotic therapies is emerging in patients requiring oxygen support. In patients with severe disease, future trials will need to consider anticoagulants and antiplatelets as additional therapies to anti‐viral and immunomodulation strategies. As thrombotic events represent disease severity, the timing of initiation of antithrombotic intervention in relation to the duration and severity of illness is likely to influence outcomes. Intravenous fibrinolytic agents have also demonstrated a rapid improvement in oxygenation, but the lack of sustained improvement necessitates a review of the treatment regimen. Critically, the bleeding risk must be proportionate to the outcomes desired. The management of COVID‐19 is likely to benefit from stratification by both severity and duration of illness, and severe disease is likely to require multiple treatment modalities.

## COMPETING INTERESTS

No competing interests were declared.

## FUNDING INFORMATION

No funding was received for this work.
